# The Prevalence and Antibiotic Susceptibility Pattern of *Salmonella typhi* among Patients Attending a Military Hospital in Minna, Nigeria

**DOI:** 10.1155/2012/875419

**Published:** 2012-09-28

**Authors:** N. U. Adabara, B. U. Ezugwu, A. Momojimoh, A. Madzu, Z. Hashiimu, D. Damisa

**Affiliations:** ^1^Department of Microbiology, Federal University of Technology, PMB 65, Minna, Nigeria; ^2^31, Artillery Brigade Medical Centre, Minna 0098, Nigeria; ^3^Department of Microbiology, Faculty of Natural Sciences, University of Jos, Jos 2084, Nigeria

## Abstract

The threat to human health posed by antibiotic-resistant bacterial pathogens is of growing concern to medical practice. This study investigated the antibiotic sensitivity pattern of *Salmonella typhi* isolated from blood specimen. One hundred blood samples were collected from suspected typhoid fever patients in 31 Artillery Brigade Medical Centre, Minna, and were analyzed for *S. typhi* while antibiotic sensitivity testing was done Kirby-Bauer method. Sixty (60.0%) samples out of the total 100 were positive for bacterial growth. The organisms isolated 2 include *Salmonella typhi*; 45 (75.0%), *Shigella*; 6 (10.0%), *E. coli*; 3 (5.0%), *Klebsiella*; 3 (5.0%), *Enterobacter*; 2 (3.3%), and *Citrobacter*; 1 (1.7%). Result of the sensitivity test showed that the isolates were resistant to all the antibiotics; ceftriaxone, cefuroxime, amoxicillin, ampicillin, ciprofloxacin, and augmentin, which are the drug of choice routinely used in the study area for the treatment of typhoid fever. They were however sensitive to chloramphenicol and ofloxacin, which, unfortunately, are not used in this study area for the treatment of typhoid fever. There appear to be multiple drug resistant (MDR) strain of *S. typhi* in the study area. These may be as a result of overdependence or uncontrolled use of the few available antibiotics and/or inaccurate or inconclusive diagnosis resulting in the development and spread of resistant strains of *S. typhi*. The study, therefore, highlights the need for a strong collaboration between the physicians and the laboratory in the choice of antibiotics for the treatment of bacterial diseases in order to discourage the development of resistant strain of bacterial pathogen.

## 1. Introduction

Typhoid fever (enteric fever) caused by the bacterium *Salmonella enteric *serovar *typhi* is an endemic disease in the tropic and subtropic. The disease is systemic and is often contracted by ingestion of food or water that is contaminated with the pathogen usually from a feco-oral source. It may, therefore, be reasonable to conclude that the occurrence of typhoid fever is an indicator of poor personal and environmental hygiene. The illness may be mild or severe but sometimes fatal. It is encountered worldwide but is primarily found in developing countries where sanitary conditions are poor [1]. Typhoid fever is now uncommon in developed countries where most occurrences are either acquired abroad or imported by emigrants [2]. With an estimatedannual incidence of 540 per 100,000 or about 17 million cases worldwide [3], the disease is considered a major public health problem. In tropical countries including Nigeria where the disease is often encountered, they account for several cases of morbidities and mortalities [4]. 

Enteric fever has continued to be a major health problem despite the use of antibiotics and the development of newer antibacterial drugs. The causative organism *Salmonella typhi* has rapidly gained resistance to antibiotics like ampicillin, ceftriaxone, and cotrimoxazole, and also to previously efficacious drugs like ciprofloxacin [5]. The emergence of antimicrobial resistance, especially the multidrug resistance to ampicillin, chloramphenicol, and cotrimoxazole, has further complicated the treatment and management of enteric fever [6]. The resistance to well-known and trusted antimicrobial agents is widely recognized as one of the greatest challenges that physicians face in the management of adult and pediatric infections [7]. 

*Salmonella typhi*, particularly the multidrug resistant (MDR) strain is relatively ubiquitous and is the cause of many community endemic and epidemic typhoid fever infections. MDR strain of *S. typhi* is of concern not only because of its resistance to available antibiotics resulting in high death rate but also because of its potential for epidemic outbreaks, which may be difficult to manage. The consequence of such outbreak will no doubt be devastating especially in developing countries where health facilities are often inadequate.

It has been opined that the initial development of resistance by *S. typhi* and most other bacterial pathogens occurred as a result of indiscriminate use of antibacterial drugs hence the need for caution in antibiotic prescription and administration. Determining the antibiotic sensitivity pattern of isolates is necessary since it will guide the physicians in making the right choice of drugs when treating patients thus ensuring quick treatment of the infection without aggravating the illness and preventing antibiotic resistance.

This study investigated the antibiotic sensitivity pattern of *Salmonella typhi* isolated from patients in Minna. 

## 2. Materials and Methods

### 2.1. Study Area

The study area is 31 Artillery Brigade Medical Centre, Minna, Niger State. The hospital is located in the Nigerian Army Cantonment situated at km 98, Suleja/Abuja road, Minna. 

### 2.2. Study Population

A total number of 100 suspected typhoid fever patients aged from 1–50 years were screened for *S. typhi* in this study. The subjects were all inhabitants of the Cantonment comprising of military personnel and civilians with a common source of drinking water. Subjects were enlisted based on informed consent.

### 2.3. Collection of Blood Samples

Five milliliters of blood was collected from each subject by venupuncture for the detection of *Salmonella typhi*. The blood samples were collected over a period of 30 days. Ten (10) samples were collected on each visit to the hospital. Sample collection was done using sterile disposable syringes and blood bottles containing anticoagulant (EDTA). The samples were quickly transported to the Microbiology Laboratory of the Federal University of Technology, Minna, where the analysis was done.

### 2.4. Preparation of Salmonella-Shigella Agar

Approximately 16 g of the dehydrated Salmonella-Shigella Agar was dissolved in 250 mL of distilled water in the preparation of the growth medium. This is in agreement with the manufacturer's directive. The mixture was brought to boil with frequent agitation until the agar has dissolved properly. It was then allowed to cool down to 50°C after which it was dispensed into petri dishes and allowed to gel. 

### 2.5. Isolation of Bacteria

Blood samples were inoculated into Salmonella-Shigella Agar (SSA) by streaking method after which the plates were incubated at 37°C for 24 hours. Isolates from the primary cultures were subcultured into fresh SSA medium to obtain pure isolates. Pure isolates were inoculated in nutrient agar slant and stored at 5°C for further characterization and identification.

### 2.6. Characterization and Identification

Pure isolates obtained on nutrient agar were identified and characterized based on colonial morphology, cultural characteristics, Gram stain reaction, and biochemical tests.

### 2.7. Antibiotic Sensitivity Testing

Antibiotic sensitivity testing was done using Kirby-Bauer method on Mueller Hinton Agar. 3.8 g of this agar was dispensed into a sterile conical flask. One hundred milliliter (100 mL) of distilled water was poured into the flask and stirred to dissolve the agar. The mixture was autoclaved and then poured into petri dishes. On gelling, the negative antibiotic sensitivity disk was introduced using sterile forceps and then incubated for 24 hours at 37°C.

## 3. Results

Out of the 100 samples investigated, 60 (60.0%) samples were positive for bacterial growth. The result of the characterization and identification of the isolates revealed that *Salmonella typhi *had the highest frequency of occurrence with 45 (75.0%) isolates followed in descending order by *Shigella* 6 (10.0%),* E. coli* 3 (5.0%), *Klebsiella* 3 (5.0%)*, Enterobacter *2 (3.3%), and *Citrobacter* 1 (1.7%), respectively (Table 1).

### 3.1. Distribution of *S. typhi* Isolated from Subjects on the Basis of Age

Out of the 45 samples that were positive for *S. typhi*. The highest frequency of occurrence was found between the ages of 1–10 years while 41–50 years had the least frequency of occurrence (Table 2).

### 3.2. Result of Antibiotic Sensitivity Test

The result of the *in vitro* antibiotic sensitivity test showed that isolates of *S. typhi* were generally resistant to ceftriaxone, cefuroxime, amoxicillin, ampicillin, ciprofloxacin, and augmentin which are the drugs of choice routinely used in the study area for the treatment of typhoid fever. They were, however, sensitive to ofloxacin and chloramphenicol even though these two antibiotics are no longer used for the treatment of typhoid fever in the study area on account of adverse reactions (Table 3).

*Enterobacter, Citrobacter, Escherichia, Klebsiella and Shigella* were all resistant to the antibiotics except amoxicillin.* Shigella* is weakly sensitive to ampicillin and strongly sensitive to ciprofloxacin, both which are not active against *S. typhi*. 

## 4. Discussion

The result of this study revealed a prevalence rate of 45.0% for typhoid fever in the study area. This finding is similar to the 46.0% prevalence rate earlier reported [8] in Tribhuvan University Teaching Hospital, Nepal, India, both of which agree with the position of the World Health Organization [9] that the vast majority of typhoid fever cases occur in Asia, Africa and Latin America where water borne diseases are highly prevalent because of inadequate supply of potable water to the public with concomitant poor environmental and personal hygiene. 

The result also revealed a high prevalence of typhoid fever (44.4%) among children within the age group (1–10) years which is also in agreement with an earlier report of 43.9% prevalence rate [10] in Cebu City, India. It is reasoned that children form the most vulnerable group in environments where inadequate water supply and poor environmental hygiene are problems because of their high level of ignorance. They are usually quick to satisfy their thirst irrespective of the water source especially if the water is apparently clean and without color. 

There was no significant difference in clinical presentation shown by the subjects as against what has been reported [11] in the editorial commentary of the February edition of Clinical Infectious Diseases from Uganda where intestinal perforation was observed among children aged between 1–5 years. In the study area, access to medical care is free which makes it easy for subjects to seek medical attention without the needless delay that often account for the pathologic damages seen in cases where access to healthcare services is constrained such as intestinal perforation.

High level of antibiotic resistance by isolates was observed to the routinely used antibiotics in the study area.this in agreement with the earlier report of a worldwide occurrence of multidrug resistance strains of *S. typhi*. This development as observed may be traceable to wrong and inaccurate diagnosis and abusive use of the available antibiotics resulting in the development and spread of multidrug resistant strains of *S. typhi*. 

Malaria is endemic in Africa and the area under study is not an exception especially during wet season when the population of mosquito vectors increases as a result of stagnant waters and bushy surroundings. The isolation of nontyphoidal bacterial agents from suspected typhoid patients calls for a review of the present approach to the diagnosis of typhoid in the study area. Improper diagnosis leads to the misuse of antibiotics by physicians which in turn lead to the development of resistance by organisms. 

The result of this study indicates that chloramphenicol and ofloxacin (which are no longer routinely used for the treatment of typhoid fever in the study area) have proved to be active against this isolates even though they were resistant to the commonly prescribed drugs. This observation that an organism that is previously resistant to a particular antibiotic may become susceptible if treatment with the antibiotic is suspended for a long time supporting what has been reported earlier [12] is interesting because of its obvious implication for public health management. However, more studies are recommended in this in this regard. 

## 5. Conclusion

The result of this study has further accentuated the growing concern about the presence of and the spread of multidrug resistant *S. typhi* thereby underscoring the need for rational application of antibiotics and other necessary interventions that will help to control the menace of antibiotic resistance. Provision of potable water, accurate laboratory diagnosis, public education, and so forth, are, therefore, recommended. Surveillance programs to monitor antimicrobial resistance patterns in other parts of the state and the entire country in general are also recommended. 

## Figures and Tables

**Table 1:** Frequency of occurrence of Bacterial isolates.

Organisms isolated	Frequency	Percentage frequency (%)
*Salmonella typhi*	45	75.0
*Shigella sp.*	6	10.0
*E. coli.*	3	5.0
*Klebsiella sp.*	3	5.0
*Enterobacter sp.*	2	3.3
*Citrobacter sp.*	1	1.7

Total	60	100

**Table 2:** Distribution of *S. typhi* isolated from subjects on the basis of age.

Age group	No investigated	No positive for *S. typhi *	Frequency of (%)
1–10	30	20	44.46
11–20	20	8	17.76
21–30	25	7	15.56
31–40	15	7	15.56
41–50	10	3	6.76

Total	100	45	100%

**Table 3:** Measurement of zone of inhibition.

Disc code (antibiotic name) (*S. typhi*)	Ave. diameter of zone of inhibition (mm)	Sensitivity pattern
Ampicillin	2.5	R
Cefuroxime	5.5	R
Ceftriaxone	5.4	R
Chloramphenicol	17.3	S
Ciprofloxacin	6.5	R
Ofloxacin	15.2	S
Amoxicillin	3.5	R
Augmentin	5.2	R

Key: S: sensitive, I: intermediate, R: resistant.
